# South Asian-Specific *MYBPC3*^Δ25bp^ Deletion Carriers Display Hypercontraction and Impaired Diastolic Function Under Exercise Stress

**DOI:** 10.3389/fcvm.2021.766339

**Published:** 2021-12-23

**Authors:** Sholeh Bazrafshan, Robert Sibilia, Saavia Girgla, Shiv Kumar Viswanathan, Megan J. Puckelwartz, Kiranpal S. Sangha, Rohit R. Singh, Mashhood Kakroo, Roman Jandarov, David M. Harris, Jack Rubinstein, Richard C. Becker, Elizabeth M. McNally, Sakthivel Sadayappan

**Affiliations:** ^1^Division of Cardiovascular Health and Disease, Department of Internal Medicine, Heart, Lung and Vascular Institute, College of Medicine, University of Cincinnati, Cincinnati, OH, United States; ^2^Center for Genetic Medicine, Feinberg School of Medicine, Northwestern University, Chicago, IL, United States

**Keywords:** *MYBPC3*, South Asians, hypertrophic cardiomyopathy, ventricular diastolic dysfunction, stress echocardiography, 25bp deletion, DOSA study, myosin binding protein-C

## Abstract

**Background:** A 25-base pair (25bp) intronic deletion in the *MYBPC3* gene enriched in South Asians (SAs) is a risk allele for late-onset left ventricular (LV) dysfunction, hypertrophy, and heart failure (HF) with several forms of cardiomyopathy. However, the effect of this variant on exercise parameters has not been evaluated.

**Methods:** As a pilot study, 10 asymptomatic SA carriers of the *MYBPC3*^Δ25bp^ variant (52.9 ± 2.14 years) and 10 age- and gender-matched non-carriers (NCs) (50.1 ± 2.7 years) were evaluated at baseline and under exercise stress conditions using bicycle exercise echocardiography and continuous cardiac monitoring.

**Results:** Baseline echocardiography parameters were not different between the two groups. However, in response to exercise stress, the carriers of Δ25bp had significantly higher LV ejection fraction (%) (CI: 4.57 ± 1.93; *p* < 0.0001), LV outflow tract peak velocity (m/s) (CI: 0.19 ± 0.07; *p* < 0.0001), and higher aortic valve (AV) peak velocity (m/s) (CI: 0.103 ± 0.08; *p* = 0.01) in comparison to NCs, and E/A ratio, a marker of diastolic compliance, was significantly lower in Δ25bp carriers (CI: 0.107 ± 0.102; *p* = 0.038). Interestingly, LV end-diastolic diameter (LVID_dia_) was augmented in NCs in response to stress, while it did not increase in Δ25bp carriers (CI: 0.239 ± 0.125; *p* = 0.0002). Further, stress-induced right ventricular systolic excursion velocity s' (m/s), as a marker of right ventricle function, increased similarly in both groups, but tricuspid annular plane systolic excursion increased more in carriers (slope: 0.008; *p* = 0.0001), suggesting right ventricle functional differences between the two groups.

**Conclusions:** These data support that *MYBPC3*^Δ25bp^ is associated with LV hypercontraction under stress conditions with evidence of diastolic impairment.

## Introduction

Mutations in sarcomere genes are a common cause of familial hypertrophic cardiomyopathy (HCM), a prevalent cardiovascular genetic disorder that affects ~1 in 200–500 asymptomatic young adults in the United States (1–11). Mutations in *MYBPC3*, which encodes cardiac myosin-binding protein-C (cMyBP-C), a thick-filament cardiac muscle protein that regulates cardiac contractility (6, 7, 12–14), are associated with ~40% of all HCM cases (9, 15), many of which present late in life (16–22). HCM is characterized by asymmetric left ventricular (LV) thickening, diastolic dysfunction, heart failure (HF), and sudden cardiac death (SCD) (4, 23, 24). Affected individuals can remain asymptomatic or present with symptoms, such as dyspnea, angina, or palpitation (9), in early or late adulthood (11, 22). Heterogeneity in the phenotypic expression of disease from *MYBPC3* genetic variation requires an understanding of early features, optimal care, and longitudinal follow-up.

**Figure 1:**
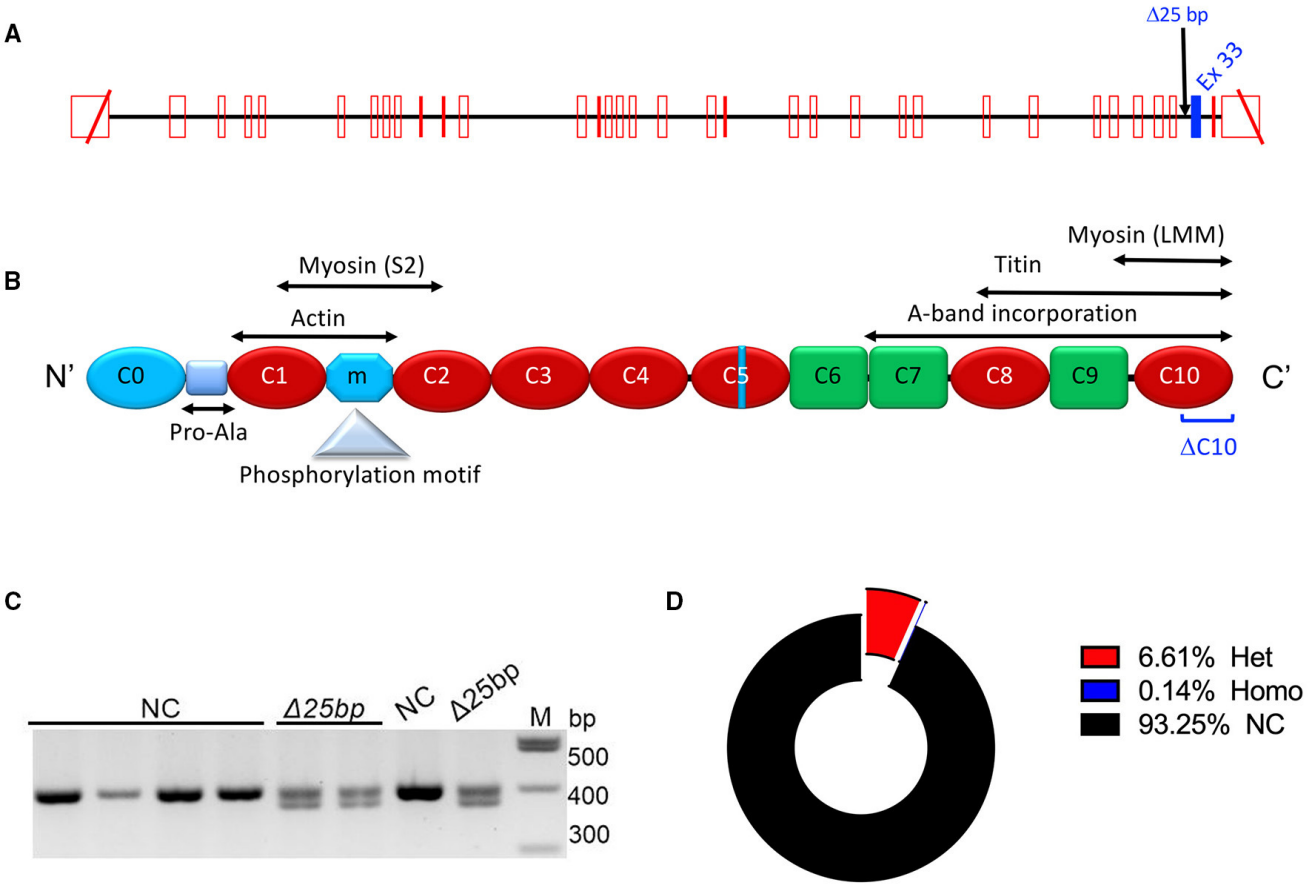
*MYBPC3*^Δ25bp^ genotyping and prevalence. **(A)** Schematic diagram of the MYBPC3 gene that includes the location of *MYBPC3*^Δ25bp^ variant in Intron 32. Exon 33 is highlighted in blue as a potential skipped exon and altered splicing exon when the *MYBPC3*^Δ25bp^ variant is pathogenic. **(B)** cMyBP-C domains are drawn with interacting partners, proline-alanine (Pro-Ala)-rich region, phosphorylation motif, and potential C10 domain that could be modified if exon 33 is altered (DC10). **(C)** Agarose gel electrophoresis of PCR-based genotyping of the *MYBPC3*^Δ25bp^ variant. **(D)** *MYBPC3*^Δ25bp^ distribution of 3,432 South Asian participants. Among carriers of the *MYBPC3*^Δ25bp^ variant, 6.61% of carriers (227, red color sector) were heterozygous (Het), and 0.14% of carriers (5, blue color sector) were homozygous (Homo). NCs, non-carriers.

The prevalence of HCM may be affected by ancestry (4, 25), but the contribution of ancestry-based genetic variants in the pathogenesis of the disease is not yet well-established. Of the South Asian (SA)-specific variants, a polymorphic 25-base pair (bp) deletion in intron 32 (Δ25bp) of *MYBPC3* is present in 4–6% (26–28) of SA individuals and a risk allele for late-onset LV dysfunction, hypertrophy, and HF with multiple forms of cardiomyopathy, such as HCM with an odds ratio of ~7 (26, 29) (Figures 1A,B). Data have shown that asymptomatic *MYBPC3*^Δ25bp^ carriers are at risk of late-onset disease progression (16, 22, 26). This variant was defined as a risk allele based on a large case-control study from 6,273 individuals belonging to 107 ethnic populations across 35 Indian states and 2,085 individuals of 63 ethnic/racial groups from 26 countries, including all five continents (26, 30). Nonetheless, in recent studies, *MYBPC3*^Δ25bp^ was reported with incomplete penetrance, and the presence of additional genetic or non-genetic risk factors may predispose carriers to cardiomyopathies (26, 27, 31). Our recent investigation has revealed no significant difference in cardiac features between the carrier and non-carrier (NC) cohorts at baseline (27). However, several individual *MYBPC3*^Δ25bp^ carriers showed increased fractional shortening at baseline, and this hyperdynamic state is often seen as an early pathological finding in HCM (27). Harper et al. identified an enriched haplotype in SAs with HCM with both *MYBPC3*^Δ25bp^ and an associated variant, *MYBPC3* c.1224-52G>A (31). However, in Harper's study, only 134 subjects out of 5,394 HCM cases were defined as SAs, and only 17 carried the *MYBPC3*^Δ25bp^ variant. We also recently identified a co-segregating novel variant, D389V (*MYBPC3*^D389V^). It was observed in ~10% of *MYBPC3*^Δ25bp^ carriers and associated with hyperdynamic cardiac features on baseline echocardiography (27). These data have led to the hypothesis that the risk of HCM is not caused by the *MYBPC3*^Δ25bp^ allele alone, but rather conferred by additional rare, pathogenic variants present on the *MYBPC3*^Δ25bp^ haplotype. This possible coinheritance of additional risk alleles changes the interpretation of the role of the *MYBPC3*^Δ25bp^ in the development of LV dysfunction and HCM. It also means that the pathogenicity of the *MYBPC3*^Δ25bp^ variant alone and in the presence of additional modifying risk factors needs further investigation and correlation, considering the growing number of SA carriers with the *MYBPC3*^Δ25bp^ variant, predisposing them to severe adverse events, such as SCD, even with the occult clinical phenotype (23).

*MYBPC3* gene variants, such as the *MYBPC3*^Δ25bp^, are generally associated with late-disease onset (16, 22, 26). Herein, we continued genetic screening of the United States (US) SA general population for the presence of *MYBPC3*^Δ25bp^ and additional rare risk alleles using next-generation sequencing (NGS) technology. To determine the pathogenicity of the *MYBPC3*^Δ25bp^ variant, we conducted an exploratory pilot study and prospectively evaluated 10 asymptomatic male carriers of the *MYBPC3*^Δ25bp^ variant and 10 age-matched NCs for the changes in cardiac function under exercise stress using submaximal bicycle stress exercise echocardiography (BSE) and continuous cardiac monitoring. We hypothesized that asymptomatic *MYBPC3*^Δ25bp^ SA carriers have detectable subclinical risk factors under exercise stress conditions that predispose this group to develop LV hypercontractility and impaired relaxation. Our results suggest that *MYBPC3*^Δ25bp^ is consistent with its role as a risk allele for LV dysfunction and cardiomyopathy in SAs.

## Methods

### Enrollment of Study Subjects: Prevalence and Genotype-Phenotype

This research study was reviewed and approved by the Institutional Review Board (IRB) of Loyola University Chicago and University of Cincinnati and was conducted in accordance with the Declaration of Helsinki. Subjects 18 years of age and older from US SA descendants from 9 countries, namely, India, Pakistan, Bangladesh, Sri Lanka, Nepal, Bhutan, Maldives, Afghanistan, and Myanmar, participated in the prevalence study (Loyola University Chicago IRB# LU207815 and 207359, Chicago, Illinois and University of Cincinnati IRB# 2016-7580, Cincinnati, OH, USA) and provided either blood or saliva samples through sample collection outreach events for detection of *MYBPC3*^Δ25bp^ variants. Additionally, carriers of *MYBPC3*^Δ25bp^ variants and age- and gender-matched NCs were recruited in the follow-up genotype-phenotype study (University of Cincinnati IRB# 2016-4948, Cincinnati, OH, USA) to perform ECG tracing and baseline transthoracic echocardiography (TTE) and BSE.

### Sample Collection and Genetic Screening of the Human *MYBPC3*^Δ25bp^ Variant

After giving written consent, blood or saliva samples of the US SA subjects were collected in community outreach events and initially screened for the detection of *MYBPC3*^Δ25bp^ variants. Blood samples (10 ml) were collected in ethylenediaminetetraacetic acid (EDTA) vacutainers (Catalog No. 367862; BD Bioscience, Woburn, MA, USA), and saliva samples (1–2 ml) were collected in sterile screw-cap transport 5-ml tubes. Saliva samples were transported in ice and stored at −20°C. Blood and saliva samples were used for human genomic DNA isolation using the QIAmp DNA Blood Mini Kit (Catalog No. 51106; Qiagen, Germantown, MD, USA). The polymerase chain reaction was used for genotyping of the *MYBPC3*^Δ25bp^ (rs36212066) variant with the forward 5′-GTT TCC AGC CTT GGG CAT AGT C-3′ and reverse 5′-GAG GAC AAC GGA GCA AAG CCC-3′ primer sequences on 2.5% agarose gel (27). After initial genotyping, we recruited carriers and NCs of the *MYBPC3*^Δ25bp^ variant for the follow-up genotype-phenotype study (27). After giving written consent, the recruited subjects provided an additional 10-ml blood sample for genotype confirmation and NGS analysis (27).

### Clinical and Cardiac Function Evaluation at Baseline and Under Exercise Stress

**Figure 2:**
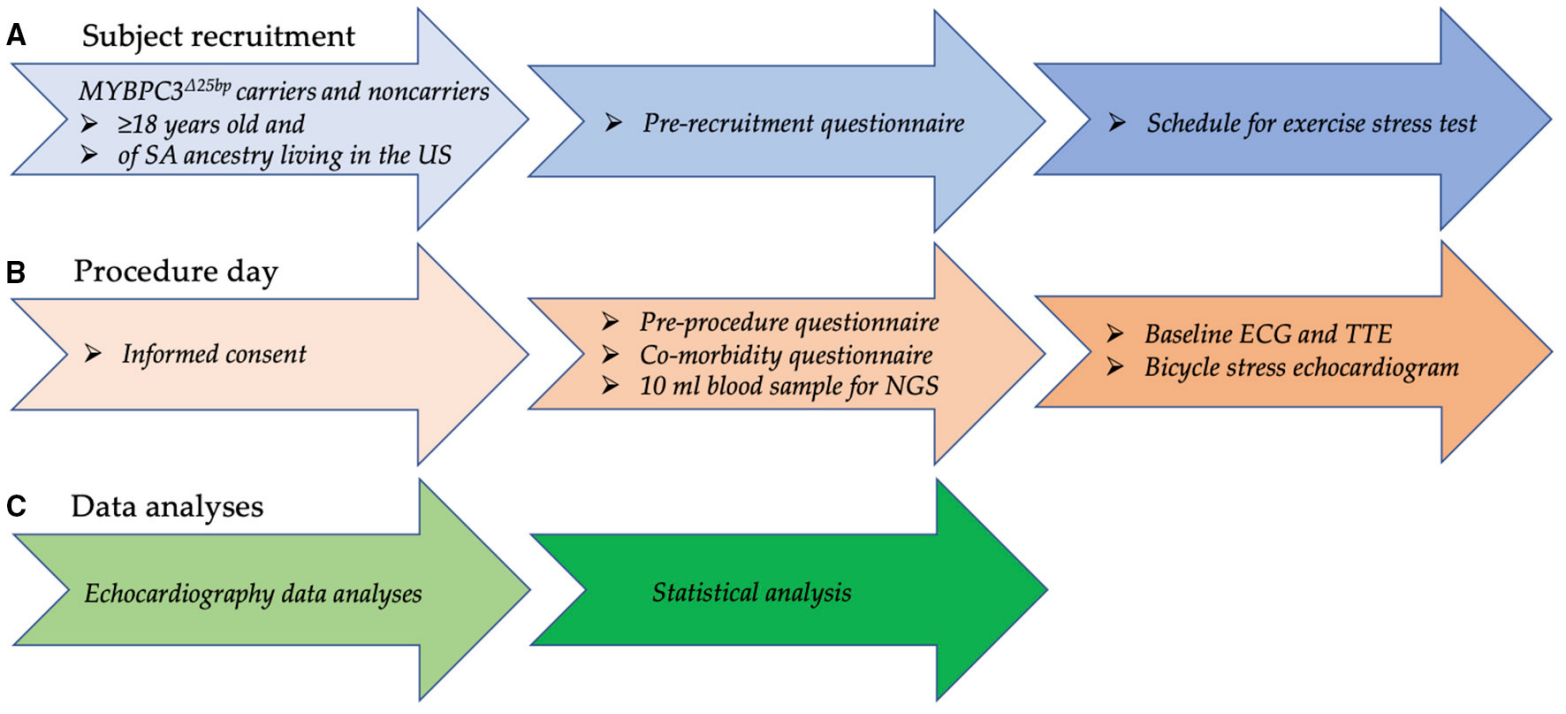
Flow chart depicting the recruitment process, employed questionnaires, and echocardiography and clinical data analyses for the genotype-phenotype study. **(A)** Carriers of *MYBPC3*^Δ25bp^ variant and age- and gender-matched non-carriers 18 years of age and older of US SA ancestry were recruited. Then, past medical and medication history was collected using a prerecruitment questionnaire, and, if no contraindication, subjects were scheduled to perform an exercise stress test using a bicycle ergometer. **(B)** On the test day, informed consent was obtained; then, a comorbidity questionnaire was used to collect comprehensive medical, medication, social, habit, and family history information from all participants. To look for other genetic modifiers via NGS testing, a 10-ml blood sample was collected. Compliance with exercise stress test instructions was evaluated via a pre-procedure questionnaire. Cardiac function was evaluated by ECG and TTE at rest and by bicycle ergometer during exercise stress testing for any detectable underlying risk factor. **(C)** While blinded, two observers independently measured echocardiography variables. Statistical significance (*p* < 0.05) was calculated using two-way ANOVA and simple linear regression, and the results were reported as mean ± SEM. SA = South Asian, NGS = next-generation sequencing. TTE, transthoracic echocardiography.

Carriers of the *MYBPC3*^Δ25bp^ variant and age- and gender-matched NCs 18 years of age and older of US SA descendants were invited to participate in the follow-up genotype-phenotype study to undergo ECG and baseline TTE and BSE, as monitored by a team cardiologist (Figure 2). Recruited subjects for the follow-up study had no uncontrolled comorbidities and no significant differences in the frequency of hypertension, diabetes mellitus, dyslipidemia, and obesity between the two groups. The subjects were instructed to abstain from alcoholic, caffeinated, tobacco products, and drugs, such as β-blockers (if applicable), for 24 h prior to stress testing. Echocardiographic images were captured by two cardiac sonographers of similar level and length of experience. Both subjects and observers, i.e., sonographers and team cardiologists, were blinded to *MYBPC3*^Δ25bp^ genotype results. Medical, medication, social, habit, and family history information of all participants were collected using a comorbidity questionnaire after giving written consent on the test day. Two additional forms, a prerecruitment questionnaire (PRQ) and a pre-procedural questionnaire (PPQ), were used for subjects who were scheduled for BSE. Before scheduling subjects for BSE testing, past medical and medication history was collected using the PRQ. Completed PRQs were then reviewed by a team cardiologist for any possible contraindications or medication modification. On the test day, subjects' compliance with BSE test instructions was evaluated using the PPQ.

Cardiac electrical activity was recorded using a standard 12-lead ECG with rhythm strips, and a Vivid E9 GE Healthcare instrument was used for TTE and BSE imaging using the 2-dimensional method in conjunction with color Doppler flow examination. Exclusion criteria for performing BSE included (1) acute myocardial infarction in the last 3 months; (2) ongoing unstable angina; (3) known obstructive left main coronary artery stenosis; (4) recent stroke or transient ischemic attack in the last 3 months; (5) acute pulmonary embolism, pulmonary infarction, or deep vein thrombosis in the last 3 months; (6) moderate-to-severe aortic stenosis with velocity over 3 m/s; (7) hypertrophic obstructive cardiomyopathy with severe resting gradient of over 3 m/s; (8) uncontrolled cardiac arrhythmia with hemodynamic compromise; (9) advanced heart block; (10) decompensated HF; (11) active endocarditis; (12) acute myocarditis or pericarditis; (13) physical disability that would preclude safe and adequate testing; (14) pregnancy; (15) mental impairment with limited ability to cooperate; (16) history of severe hypertension (resting systolic blood pressures >200 or diastolic blood pressures 110 mm Hg) or uncontrolled diabetes mellitus; and (17) uncorrected anemia, electrolyte imbalance or hyperthyroidism. Subjects were provided with the instructions for BSE preparation prior to the test that included (1) fasting for 4 h, (2) refraining from caffeinated and decaffeinated coffee and tea, colas, soft drinks, and chocolate for 24 h, and (3) refraining from smoking and other nicotine-containing products for 12 h.

Moreover, target heart rate (THR) was calculated for BSE as 220 – (0.85 × subject's age). Then, the subjects began cycling at a resistance of 15 Watts (W), increasing every 3 min by additional resistance of 15 W until the endpoint. Heart rate (HR), blood pressure, and oxygen saturation were monitored and documented at each minute of exercise. Endpoints of the test were one of the following: (1) achievement of THR; (2) significant angina; (3) significant shortness of breath; (4) significant ischemic changes, such as ST-segment elevations in ECG; (5) hypertension (>240 mmHg systolic blood pressure/ 120 mmHg diastolic blood pressure); (6) hypotension >15 mmHg decline in systolic blood pressure from baseline; (7) significant arrhythmia/aortic valve (AV) block; (8) new or worsening wall-motion abnormalities; (9) drop of oxygen saturation below 90%; (10) intolerable symptoms; (11) upon subject's request to stop; or (12) development of dynamic LV outflow tract (LVOT) gradient of >4.5 m/s.

Finally, two observers independently measured echocardiography variables under the guidance of team cardiologists. The measurements of one observer were used for the analysis in this study, except LV ejection fraction (LVEF) for which the average value measured by two observers was used. The Biplane Simpson method was used to measure LVEF and LV fractional shortening.

### Next-Generation Sequencing

A panel of 174 genes related to cardiovascular diseases was DNA sequenced to identify the presence of any genetic variants using the TruSight Cardio Kit (Catalog No. FC-141-1011; Illumina, San Diego, CA, USA) on an Illumina MiSeq with 150-base pair paired-end reads, as described previously (27). Burrows-Wheeler alignment (BWA) and Genome Analysis Toolkit haplotype caller were applied as described in MegaSeq generate variant call files (VCFs) (32). Variants were excluded if they met any of the following criteria: biallelic balance >0.75; quality score <30; depth of coverage >360; strand bias more than −0.01, and mapping quality zero reads ≥10. Variant ranking and prioritization: variants were annotated using SnpEFF. HIGH and MODERATE variants were scored using Polyphen, Genomic Evolutionary Rate Profiling, and Sorting Intolerant From Tolerant, to predict pathogenicity. All variants were ranked by minor allele frequency (MAF) based on data from public databases (ExAc). All variants were crosschecked against ClinVAR. The American College of Medical Genetics guidelines (ACMG) were used for determining pathogenicity (33). Sanger sequencing was applied for validation using individual primers.

### Statistical Analyses

The prevalence of genotype results was reported as a percentage. Descriptive statistics were reported in frequency tables to compare cardiac phenotype outcomes by genetic variants and by demographics. The chi-square statistics and unpaired *t*-test were used for categorical parameters and numerical variables at baseline, respectively. Regression analysis was used to compare differences in slopes between the groups, and a two-way ANOVA was performed to estimate the effect of exercise stress and genotype on cardiac phenotype continuous outcomes. All analyses were reported as mean ± SEM at a significance level of 0.05, and 95% CIs and *p*-values were reported using GraphPad Prism (version 8.4.3).

## Results

### Increased Prevalence of *MYBPC3*^Δ25bp^ Variant Among US SAs

We previously reported the frequency of *MYBPC3*^Δ25bp^ variant carriers to be 6% among the US SA population (27). Cumulative screening to date, which could be subject to ascertainment bias, estimated a slightly higher prevalence of *MYBPC3*^Δ25bp^ at 6.75%. Of 3,432 participants, 232 (6.75%) were carriers of the *MYBPC3*^Δ25bp^ variant, i.e., 227 (6.61%) heterozygous and 5 (0.14%) homozygous (Figures 1C,D).

### Absence of Second Genetic Variants by NGS

To assess the presence of any additional pathogenic variants, apart from the *MYBPC3*^Δ25bp^ variant, NGS was performed on a 172 gene cardiovascular panel (TruSight Cardio Sequencing Kit, Illumina), and no pathogenic, or likely pathogenic, variants were identified in the present cohort (27). None of the 20 subjects included in the current study was found to carry the previously reported, potentially pathogenic *MYBPC3*^−52^ allele (c.1224-52G>A) (31) or the novel *MYBPC3*^D389V^ variant (27). We also did not identify any additional modifying loci in *MYBPC3* as no other rare variants were identified in the coding region that occurred in more than one cohort subject. Lastly, among the 172 genes of the TruSight Panel, we only identified one pathogenic variant in one subject who carried an APOA4 variant in one of the 10 variant carriers.

### *MYBPC3*^Δ25bp^ Was Associated With Hypercontraction Under Exercise

To test the hypothesis that asymptomatic *MYBPC3*^Δ25bp^ US SA carriers have detectable subclinical risk factors under exercise stress conditions, we conducted an exploratory pilot study to evaluate asymptomatic *MYBPC3*^Δ25bp^ US SA carriers compared to controls for subclinical cardiac changes under exercise stress conditions (Figure 2). We prospectively studied 20 male subjects of US SA ancestry, including 10 carriers of the *MYBPC3*^Δ25bp^ variant (52.9 ± 2.14 years) and 10 age- and gender-matched NCs (50.1 ± 2.7 years), for any changes in cardiac function at baseline prior to exercise and under exercise stress using bicycle exercise echocardiography and continuous cardiac monitoring (Tables 1, 2). The two groups (*MYBPC3*^Δ25bp^ carriers and NCs) showed no significant difference in body surface area, body mass index, and baseline mean arterial pressure (MAP) and HR, and no difference in the frequency of comorbidities, such as hypertension, diabetes mellitus, and dyslipidemia (Table 1). Similarly, monitored exercise MAP and HR were similar in both groups (Table 2). All 20 subjects were able to complete at least 15 min of exercise at 75 W equivalent to 4.9 METs, while 4 subjects completed 30 min of exercise at 150 W equivalent to 8.5 METs.

**Table 1:** Demographics, baseline echocardiography parameters, and clinical characteristics of 20 South Asian subjects who participated in a bicycle exercise study.

**Variable**	**Total**	***n***	**NC**	***n***	***MYBPC3* ^**Δ25*bp***^**	***n***	***P*-value**
	***n* (%), mean ± SEM**		***n* (%), mean ± SEM**		***n* (%), mean ± SEM**		
Male (%)	20(100)	20	10(100)	10	10(100)	10	-
Age (years)	51.50 ± 1.71	20	50.1 ± 2.70	10	52.90 ± 2.14	10	0.427
BMI (kg/m^2^)	26.15 ± 0.611	20	26.23 ± 0.58	10	26.06 ± 1.11	10	0.893
BSA (m^2^)	1.95 ± 0.037	20	1.98 ± 0.04	10	1.91 ± 0.06	10	0.301
MAP (mmHg)	101.1 ± 2.00	20	98.70 ± 2.78	10	103.5 ± 2.82	10	0.244
HR (bpm)	74.65 ± 1.96	20	76.90 ± 2.42	10	72.40 ± 3.03	10	0.262
**Echocardiographic parameters**							
LVID_dia_ (cm)	4.06 ± 0.10	20	3.97 ± 0.15	10	4.16 ± 0.13	10	0.376
LVID_s_ (cm)	2.78 ± 0.08	20	2.69 ± 0.10	10	2.87 ± 0.14	10	0.331
LVEF (%)	53.65 ± 1.38	20	53.59 ± 1.81	10	53.70 ± 2.17	10	0.969
LVFS (%)	31.38 ± 0.99	20	31.55 ± 0.91	10	31.21 ± 1.82	10	0.867
IVS (cm)	0.94 ± 0.04	20	0.96 ± 0.07	10	0.93 ± 0.03	10	0.71
LVPW (cm)	0.88 ± 0.03	20	0.93 ± 0.03	10	0.83 ± 0.04	10	0.111
IVS/LVPW ratio	1.09 ± 0.05	20	1.04 ± 0.08	10	1.14 ± 0.05	10	0.351
LAV 4C Mod	31.75 ± 2.95	18	30.25 ± 4.69	8	32.95 ± 3.94	10	0.663
LVOT peak velocity (m/s)	0.79 ± 0.03	19	0.76 ± 0.05	9	0.82 ± 0.03	10	0.424
AV peak velocity (m/s)	0.98 ± 0.03	19	0.96 ± 0.06	9	1.01 ± 0.04	10	0.551
E/A ratio	1.15 ± 0.08	20	1.10 ± 0.13	10	1.20 ± 0.11	10	0.596
Average E/e' ratio	7.18 ± 0.46	20	7.20 ± 0.49	10	7.16 ± 0.82	10	0.973
Average e'/a' ratio	1.01 ± 0.08	20	0.90 ± 0.10	10	1.13 ± 0.13	10	0.204
RV s' (m/s)	0.097 ± 0.003	20	0.094 ± 0.004	10	0.09 ± 0.004	10	0.458
LV s' average (m/s)	0.07 ± 0.002	20	0.07 ± 0.003	10	0.07 ± 0.003	10	0.437
TAPSE (cm)	2.21 ± 0.12	19	2.40 ± 0.23	9	2.04 ± 0.10	10	0.166
**Clinical characteristics**							
HTN (%)	5(25)	20	2(20)	10	3(30)	10	>0.999
DM (%)	5(25)	20	3(30)	10	2(20)	10	>0.999
DLP (%)	8(40)	20	4(40)	10	4(40)	10	>0.999

*Statistical significance (p < 0.05) was calculated using unpaired t-test for numerical variables and Fisher's exact test for categorical variables*.

*The results were reported as mean ± SEM. NCs, non-carriers; MYBPC3^Δ2bp^, A 25-base pair deletion in cardiac myosin-binding protein C3; SEM, standard error of mean; BMI, body mass index; BSA, body surface area; MAP, mean arterial pressure; HR, heart rate; LVID_dia_, left ventricular internal diameter in diastole; LVID_s_, left ventricular internal diameter in systole; LVEF, left ventricular ejection fracture; LVFS, left ventricular fractional shortening; IVS, interventricular septum; LVPW, left ventricular posterior wall; LAV 4C Mod, left atrial volume four-chamber method of disc; LVOT, left ventricular outflow tract; AV, aortic valve; E/A ratio, ratio of early diastole transmitral peak velocity flow to late diastole peak velocity flow; E/e′ ratio, ratio of early transmitral peak velocity flow to early diastolic mitral annulus velocity; e′/a′ ratio, ratio of early diastolic mitral annulus velocity to late diastolic mitral annulus velocity; RV, right ventricle; s′, peak systolic annular velocity; LV, left ventricle; TAPSE, tricuspid annular plane systolic excursion; HTN, hypertension; DM, diabetes mellitus; DLP, dyslipidemia*.

**Table 2:** Clinical and bicycle exercise echocardiography parameters of 20 South Asian subjects.

**Stress stage (W)**		**0**		**15**		**30**		**45**		**60**		**75**		**90**		**105**		**120**		
**Variables**		**Mean ± SEM**	***n***	**Mean ± SEM**	***n***	**Mean ± SEM**	***n***	**Mean ± SEM**	***n***	**Mean ± SEM**	***n***	**Mean ± SEM**	***n***	**Mean ± SEM**	***n***	**Mean ± SEM**	***n***	**Mean ± SEM**	***n***	***P*-value**
MAP (mmHg)	NC	98.70 ± 2.78	10	107.93 ± 2.37	10	110.1 ± 3.55	10	110.03 ± 3.73	10	109.2 ± 3.74	10	114.43 ± 2.94	10	114.75 ± 3.56	8	119.62 ± 4.96	8	117.33 ± 7.19	6	0.412
	*MYBPC3* ^Δ25*bp*^	103.46 ± 2.82	10	109.92 ± 3.44	9	108.77 ± 2.74	9	112.53 ± 3.40	10	111.66 ± 4.34	10	112.76 ± 5.89	10	113.14 ± 6.31	9	123.44 ± 5.89	6	121.13 ± 4.57	5	
HR (bpm)	NC	76.90 ± 2.42	10	92.5 ± 1.8	10	98 ± 2.04	10	105.2 ± 2.4	10	112.5 ± 2.6	10	118 ± 2.02	10	125.75 ± 3.39	8	137.12 ± 4	8	146.83 ± 5.38	6	0.21
	*MYBPC3* ^Δ25*bp*^	72.40 ± 3.03	10	91.2 ± 2.56	10	96.7 ± 2.92	10	103.5 ± 2.60	10	111.3 ± 3.34	10	119.5 ± 3.25	10	125.11 ± 3.63	9	132.83 ± 4.93	6	143.66 ± 4.69	6	
LVID_dia_ (cm)	NC	3.97 ± 0.15	10	4.4 ± 0.08	9	4.52 ± 0.11	9	4.56 ± 0.11	9	4.29 ± 0.12	9	4.59 ± 0.15	7	4.57 ± 0.1	6	4.59 ± 0.12	5	4.44 ± 0.13	4	0.0002
	*MYBPC3* ^Δ25*bp*^	4.16 ± 0.13	10	4.14 ± 0.13	10	4.17 ± 0.09	10	4.15 ± 0.08	9	4.18 ± 0.12	9	4.34 ± 0.15	7	4.32 ± 0.07	7	4.31 ± 0.16	6	4.01 ± 0.21	4	
LVID_s_ (cm)	NC	2.69 ± 0.1	10	2.81 ± 0.07	9	2.77 ± 0.1	9	2.65 ± 0.09	9	2.47 ± 0.08	9	2.67 ± 0.05	7	2.53 ± 0.09	6	2.45 ± 0.09	5	2.36 ± 0.07	4	0.06
	*MYBPC3* ^Δ25*bp*^	2.87 ± 0.14	10	2.68 ± 0.1	10	2.63 ± 0.07	10	2.48 ± 0.05	9	2.37 ± 0.1	9	2.54 ± 0.07	7	2.39 ± 0.07	7	2.39 ± 0.07	6	2.24 ± 0.12	4	
LVEF (%)	NC	53.59 ± 1.81	10	55.26 ± 1.60	10	58.14 ± 2.09	10	59.99 ± 2.17	10	61.53 ± 2.12	10	64.03 ± 2.27	10	62.86 ± 2.83	7	61.83 ± 3.05	7	61.37 ± 3.39	5	<0.0001
	*MYBPC3* ^Δ25*bp*^	53.7 ± 2.17	10	58.26 ± 1.62	10	60.76 ± 1.69	10	63.07 ± 1.63	10	65.91 ± 1.71	10	67.65 ± 1.09	9	68.82 ± 1.48	8	70.19 ± 0.75	7	71.43 ± 0.58	4	
LVFS (%)	NC	31.55 ± 0.91	10	35.75 ± 0.95	9	38.79 ± 1.13	9	42.02 ± 1.03	9	42.19 ± 1.55	9	42.9 ± 1.81	7	44.62 ± 1.29	6	45.83 ± 2.33	5	46.39 ± 2.66	4	0.218
	*MYBPC3* ^Δ25*bp*^	31.2 ± 1.82	10	35.98 ± 1.68	10	36.98 ± 1.41	10	40.15 ± 1.39	9	43.44 ± 2.14	9	41.24 ± 1.71	7	44.51 ± 1.9	7	43.72 ± 2.03	6	43.93 ± 1.53	4	
LVOT peak velocity (m/s)	NC	0.76 ± 0.05	9	0.94 ± 0.06	9	0.91 ± 0.04	9	0.97 ± 0.06	10	1.00 ± 0.07	10	1.06 ± 0.04	8	1.13 ± 0.03	6	1.18 ± 0.04	5	1.33 ± 0.11	4	<0.0001
	*MYBPC3* ^Δ25*bp*^	0.82 ± 0.03	10	1.12 ± 0.06	10	1.08 ± 0.06	9	1.17 ± 0.06	10	1.34 ± 0.08	10	1.29 ± 0.07	10	1.36 ± 0.08	9	1.39 ± 0.07	7	1.49 ± 0.14	4	
AV peak velocity (m/s)	NC	0.96 ± 0.06	9	1.15 ± 0.06	9	1.09 ± 0.08	10	1.23 ± 0.09	9	1.30 ± 0.09	9	1.35 ± 0.07	8	1.43 ± 0.07	6	1.41 ± 0.13	4	1.61 ± 0.14	3	0.012
	*MYBPC3* ^Δ25*bp*^	1.01 ± 0.04	10	1.2 ± 0.06	10	1.25 ± 0.08	8	1.31 ± 0.07	10	1.38 ± 0.07	10	1.45 ± 0.07	10	1.49 ± 0.08	8	1.6 ± 0.06	8	1.75 ± 0.07	4	
E/A	NC	1.10 ± 0.13	10	1.16 ± 0.06	10	1.15 ± 0.08	10	0.97 ± 0.06	9	1.10 ± 0.06	10	0.96 ± 0.09	9	1.05 ± 0.03	6	1.10 ± 0.04	6	1.15 ± 0.20	3	0.03
	*MYBPC3* ^Δ25*bp*^	1.2 ± 0.11	10	0.9 ± 0.05	9	1.00 ± 0.09	9	1.01 ± 0.07	10	1.00 ± 0.1	9	0.96 ± 0.07	7	0.89 ± 0.09	5	0.77 ± 0.05	3	1.03 ± 0.27	2	
E/e' average	NC	7.20 ± 0.49	10	7.01 ± 0.50	9	7.31 ± 0.56	10	6.86 ± 0.63	8	7.25 ± 0.31	10	7.84 ± 0.63	7	8.38 ± 0.72	5	7.52 ± 0.40	6	8.81 ± 0.53	2	0.068
	*MYBPC3* ^Δ25*bp*^	7.16 ± 0.82	10	7.44 ± 0.98	9	6.27 ± 0.74	7	7.32 ± 0.80	9	7.58 ± 0.87	7	6.84 ± 0.92	6	6.19 ± 0.89	4	6.62 ± 0.47	3	6.11 ± 0.84	2	
RV s' (m/s)	NC	0.095 ± 0.004	10	0.107 ± 0.007	10	0.109 ± 0.007	10	0.121 ± 0.006	8	0.130 ± 0.01	10	0.139 ± 0.009	9	0.16 ± 0.009	7	0.158 ± 0.01	7	0.172 ± 0.009	6	0.35
	*MYBPC3* ^Δ25*bp*^	0.1 ± 0.005	10	0.12 ± 0.004	8	0.117 ± 0.005	8	0.131 ± 0.005	8	0.132 ± 0.008	8	0.139 ± 0.009	8	0.152 ± 0.009	6	0.157 ± 0.01	5	0.175 ± 0.014	3	
LV s' average (m/s)	NC	0.07 ± 0.004	10	0.08 ± 0.003	10	0.08 ± 0.004	10	0.08 ± 0.003	9	0.09 ± 0.003	10	0.101 ± 0.004	9	0.101 ± 0.006	8	0.114 ± 0.009	5	0.118 ± 0.014	3	0.557
	*MYBPC3* ^Δ25*bp*^	0.07 ± 0.003	10	0.08 ± 0.002	9	0.08 ± 0.003	9	0.09 ± 0.004	9	0.09 ± 0.003	8	0.103 ± 0.005	9	0.106 ± 0.005	7	0.107 ± 0.004	6	0.112 ± 0.006	4	
TAPSE (cm)	NC	2.40 ± 0.23	9	2.34 ± 0.19	9	2.31 ± 0.13	9	2.42 ± 0.14	9	2.47 ± 0.16	9	2.53 ± 0.22	7	2.63 ± 0.13	6	2.44 ± 0.15	5	2.53 ± 0.13	4	0.065
	*MYBPC3* ^Δ25*bp*^	2.04 ± 0.1	10	2.27 ± 0.13	10	2.38 ± 0.16	9	2.55 ± 0.13	10	2.68 ± 0.21	9	2.81 ± 0.1	7	2.52 ± 0.18	3	2.81 ± 0.23	6	3.71 ± 0.00	1	

*Statistical significance (p < 0.05) was calculated using two-way ANOVA. The results were reported as mean ± SEM. NCs, non-carriers; MYBPC3^Δ25bp^, A 25-base pair deletion in cardiac myosin binding protein C3; SEM, standard error of mean; MAP, mean arterial pressure; HR, heart rate; LVID_dia_, left ventricular internal diameter in diastole; LVID_s_, left ventricular internal diameter in systole; LVEF, left ventricular ejection fracture; LVFS, left ventricular fractional shortening; LVOT, left ventricular outflow tract; AV, aortic valve; E/A ratio, ratio of early diastole transmitral peak velocity flow to late diastole peak velocity flow; E/e′ ratio, ratio of early transmitral peak velocity flow to early diastolic mitral annulus velocity; RV, right ventricle; s′, peak systolic annular velocity; LV, left ventricle; TAPSE, tricuspid annular plane systolic excursion*.

**Figure 3:**
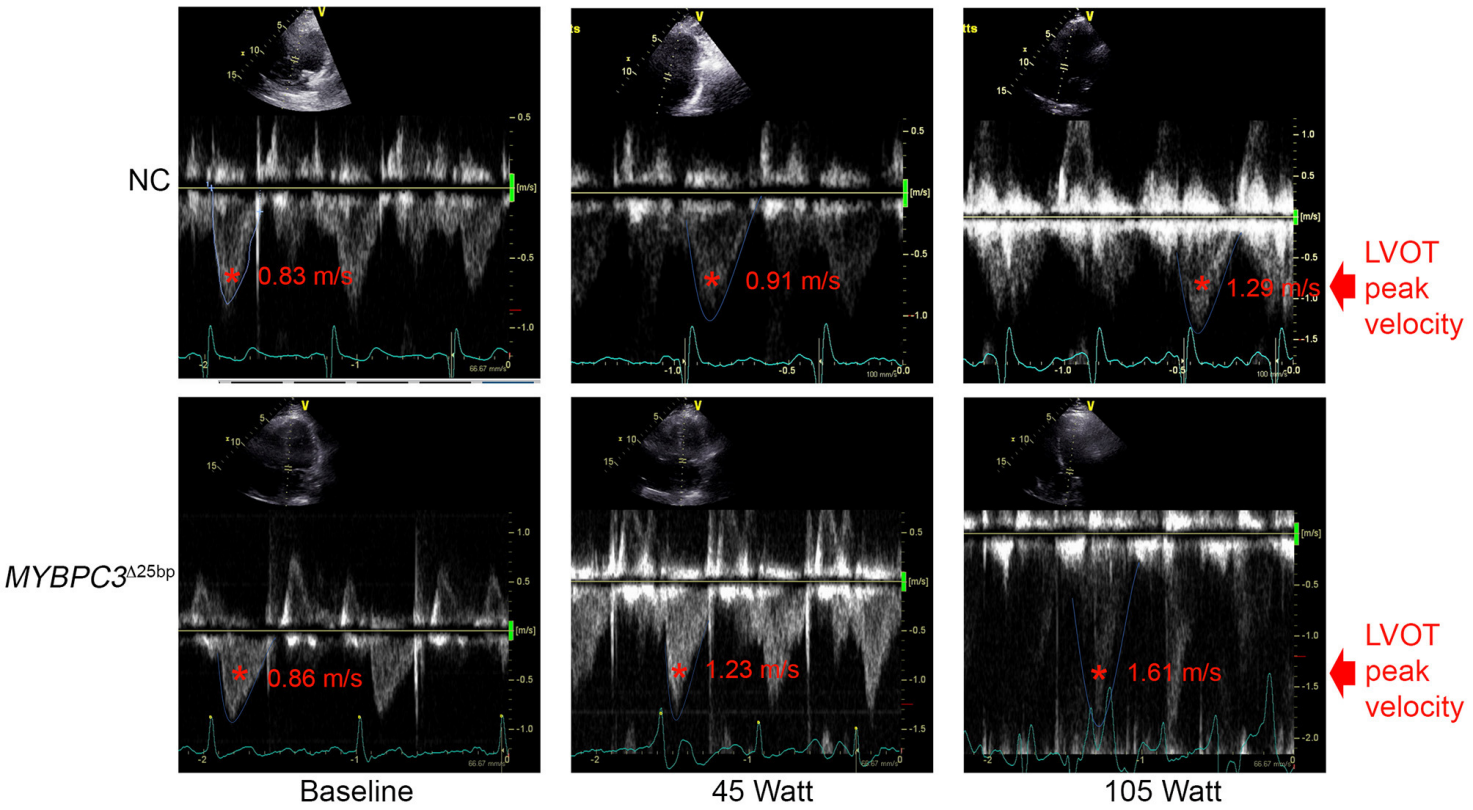
Spectral Doppler data depict LVOT peak velocity. Representative apical five-chamber view at baseline, 45 (W), and 105 (W) in a NC (upper panel) subject and a *MYBPC3*^Δ25bp^ variant carrier (lower panel). NC, non-carriers.

**Figure 4:**
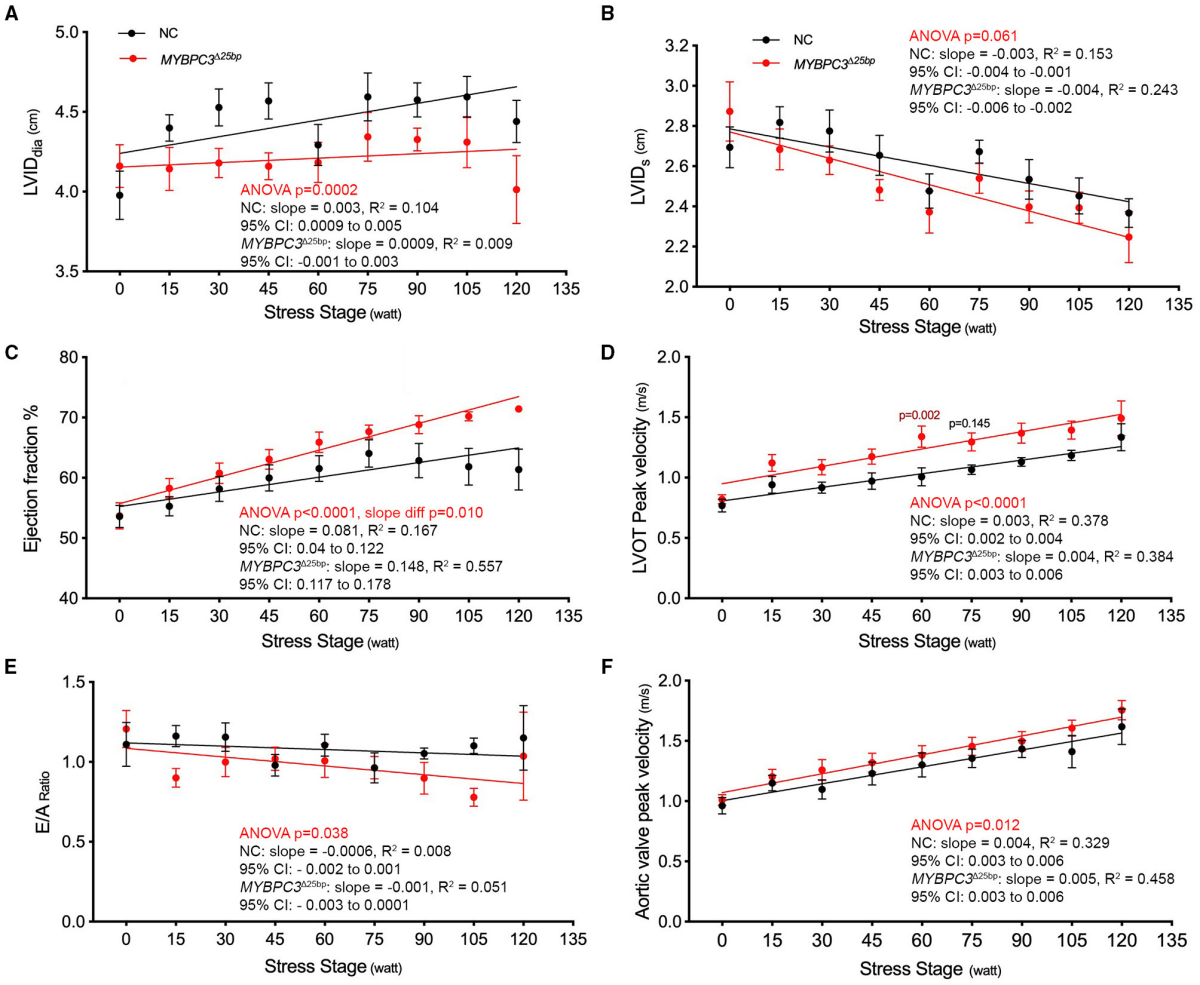
Prevalent South Asian-specific *MYBPC3*^Δ25bp^ variant is associated with hypercontraction and impaired relaxation under exercise stress (10 NCs vs. 10 *MYBPC3*^Δ25bp^ carriers). **(A)** A significant difference in LVID_dia_ is observed between carriers of the *MYBPC3*^Δ25bp^ variant and NCs (Two-way ANOVA, *p* = 0.0002; CI: 0.239 ± 0.125). In response to stress, LVID_dia_ increases significantly from baseline in NCs, whereas it does not significantly change in *MYBPC3*^Δ25bp^ carriers. **(B)** The graph depicts a non-significant difference between *MYBPC3*^Δ25bp^ variant carriers and NCs in LVID_s_ in response to stress (Two-way ANOVA, *p* = 0.061; CI: 0.09 ± 0.09). In addition, carriers of the *MYBPC3*^Δ25bp^ variant show significantly higher **(C)** ejection fraction (CI: 4.57 ± 1.93) and **(D)** LVOT peak velocity (CI: 0.197 ± 0.069) compared to NCs (Two-way ANOVA, *p* < 0.0001, both). **(E)** With exercise, the average E/A ratio shows a significant difference between *MYBPC3*^Δ25bp^ carriers and NCs (Two-way ANOVA, *p* < 0.038; CI: 0.107 ± 0.102). **(F)** The graph depicts significantly higher aortic valve peak velocity in *MYBPC3*^Δ25bp^ variant carriers compared to NCs (Two-way ANOVA, *p* < 0.012; CI: 0.103 ± 0.081). LVID_dia_, left ventricular internal diameter in diastole; LVID_s_, left ventricular internal diameter in systole; LVOT, left ventricular outflow tract; NC, non-carriers.

While baseline echocardiography parameters were not different between the two groups (*MYBPC3*^Δ25bp^ carriers and NCs) (Table 1), significant differences came to light under exercise stress condition in the following parameters: LV end-diastolic diameter (LVID_dia_), LVEF, LVOT and AV peak velocities (pv), and the ratio of early to late ventricular filling velocity (E/A ratio) (Table 2, Figures 3, 4). Stress-induced LVID_dia_ augmented in NCs, while it did not increase in *MYBPC3*^Δ25bp^ carriers with a significant difference between the two groups (CI: 0.239 ± 0.125; *p* = 0.0002), indicative of impaired relaxation and diastolic impairment. Strikingly, the estimated effect of exercise stress and genotype showed that *MYBPC3*^Δ25bp^ carriers had significantly higher LVEF (%) (CI: 4.57 ± 1.93; *p* < 0.0001), higher LVOT pv (m/s) (CI: 0.197 ± 0.069; *p* < 0.0001), and higher AV pv (m/s) (CI: 0.103 ± 0.081; *p* = 0.01) in comparison to NCs. This was consistent with the findings that stress-induced LVID_dia_ increase was significantly muted in carriers during exercise, as compared to NCs (CI: 0.239 ± 0.125; *p* = 0.0002). Further, E/A ratio, a marker of ventricular diastolic compliance, was significantly lower in carriers as compared to NCs (CI: 0.107 ± 0.102; *p* = 0.038) and the ratio of early transmitral peak velocity flow to early diastolic mitral annulus velocity (E/e' ratio), which showed a non-significant difference between the groups (CI: 0.738 ± 0.795; *p* = 0.068). Although stress-induced right ventricular systolic excursion velocity (s') was increased similarly in both groups, tricuspid annular plane systolic excursion (cm) increased more in carriers (slope: 0.008; *p* = 0.0001) from the baseline, consistent with right ventricle functional differences. These findings are indicative of LV hypercontractility among asymptomatic carriers of the *MYBPC3*^Δ25bp^ variant under exercise stress conditions and evidence of diastolic impaired relaxation at high workloads, suggesting that *MYBPC3*^Δ25bp^ is an independent risk allele with subclinical pathology prior to late-onset LV dysfunction (16, 22) in the US SA population.

## Discussion

The present study evaluated the exercise response in asymptomatic *MYBPC3*^Δ25bp^ SA carriers to determine the presence of any subclinical features. Overall, our findings determined the presence of hyperdynamic manifestations under exercise conditions in asymptomatic *MYBPC3*^Δ25bp^ variant carriers of US SA ancestry as compared to NCs who were presented with significantly higher EF, LVOT, and AV peak velocities, and impaired relaxation presented with a significant difference in the LVID_dia_ and E/A ratio, but the non-significant difference in the average E/e' ratio. Hyperdynamic features and evidence of cardiac dysfunction were detected by echocardiography in *MYBPC3*^Δ25bp^ carriers. These findings are also in line with the phenotype of genotype-positive individuals at stage 1 or those with non-hypertrophic HCM. These individuals can remain asymptomatic or present with subtle echocardiographic phenotype, such as diastolic dysfunction (34–36). In stage 2, however, individuals present with hypertrophy and LV hyperdynamic status (36), whereas *MYBPC3*^Δ25bp^ variant carriers in the current study were non-hypertrophic, but did show hyperdynamic phenotype under exercise conditions. As shown in the regression lines, NCs and carriers responded differently to the stress stage, the independent variable, as resistance increased. Starting at 75 W equiv. 4.9 METs, the regression line showed no increase in EF% in NCs after 15 min of exercise. This finding can be explained by the Frank-Starling law, which holds that an increase in workload and venous returns stretches myocardial muscle fibers and increases preload (end-diastolic volume), stroke volume, and, ultimately, cardiac output until maximum capacity is reached. However, the weak increase in LVID_dia_ from the baseline, as indicated in the regression line for carriers (Figure 4A), could be a contributing factor in maintaining an upward trend in EF% in carriers after 75 w since lower LVID_dia_ can contribute to lower LV diastolic volume (LVEDV) as resistance augments. However, further studies with an adequate number of subjects are needed to decisively explain this observed phenotypic feature. Collectively, then, these data suggest that exercise stress itself, as a secondary risk factor, potentially triggered hyperdynamic phenotype in asymptomatic *MYBPC3*^Δ25bp^ carriers, as compared to NCs, considering that the hemodynamic parameters of MAP and HR did not differ between the two groups (*MYBPC3*^Δ25bp^ variant carriers and NCs).

Previous studies have examined the distribution (26, 27, 37, 38) and clinical correlations (26, 27, 31, 39) of the *MYBPC3*^Δ25bp^ variant, noting, importantly, that most of these studies were conducted in individuals of SA ancestry. In the first report (2003), the *MYBPC3*^Δ25bp^ variant was initially detected in two affected Indian families. Then, it was reported in 16 of 229 unrelated healthy SA individuals and present in 3.8% of the general population, confirming variability in disease penetrance (29). In the next case-control study with a multi-ethnic population of 6,273 individuals in the SA Diaspora, the carrier frequency of the *MYBPC3*^Δ25bp^ variant was reported to be 4% (26). Simonson et al. (38) and Bashyam et al. (37) reported 8 and 6%, respectively, in their studies. In 2018, we determined 6% prevalence in a sample taken from individuals of SA ancestry living in the United States (27). However, *MYBPC3*^Δ25bp^ is largely associated with incomplete penetrance and delayed onset (26, 27, 29). In the current study, we identified a slightly higher prevalence at 6.75%, but given the nature of our study, this result may have been influenced by ascertainment. Collectively, these data support that *MYBPC3*^Δ25bp^ is a common variant and that its distribution is associated with ethnicity (4, 25). Furthermore, our interpolation indicates that a prevalent SA-specific *MYBPC3*^Δ25bp^ variant predisposes an estimated ~100 million people of SA ancestry worldwide (26) to such adverse cardiac events as cardiomyopathies, arrhythmias, HF, and SCD.

Previously, we reported no significant difference in clinical characteristics, ECG, or echocardiographic parameters between US SA carriers of the *MYBPC3*^Δ25bp^ variant and NCs at rest, except for LV fractional shortening (27), which was slightly higher in carriers as compared to NCs (*p* = 0.04), suggesting a minimal effect of the *MYBPC3*^Δ25bp^ variant on the development of HCM phenotype. Compared to the current study, prior analyses included more individuals (n = 47 carriers) who were younger (47.6 years) and, importantly, included both men and women (27). Both sex inclusion and age may have reduced the ability to detect a hyperdynamic state, which we discovered upon exercise stress. Many affected individuals could also remain asymptomatic until late adulthood (16, 22). Therefore, early detection of any subtle clinical phenotype and/or secondary contributing risk factors triggering symptoms in the carrier population, as we have suggested in our findings, is potentially relevant clinically. In the prior study, we also identified a second co-segregating novel variant (D389V) identified in ~10% of *MYBPC3*^Δ25bp^ carriers associated with hyperdynamic cardiac features on echocardiogram as a potential secondary modifying factor for HCM (27). In this current investigation, however, no *MYBPC3*^Δ25bp^ subjects with the D389V variant were included, meaning that the findings herein cannot be attributed to D398V. As previously noted, Harper et al. separately identified a haplotype in SAs with HCM with both the *MYBPC3*^Δ25bp^ variant and a potentially pathogenic variant, *MYBPC3* c.1224-52G>A (31). None of the members of our *MYBPC3*^Δ25bp^ cohort were, in fact, found to carry the variant identified by Harper et al. Altogether, we propose that *MYBPC3*^Δ25bp^ is still a valid risk variant in the etiology of HCM and that it arises from a secondary risk factor, namely, exercise stress, and manifests as hypercontraction and impaired relaxation associated with late-onset LV dysfunction.

Exercise stress echocardiography is a standard clinical assessment and diagnostic armamentarium of HCM to assess exercise-induced LVOT obstruction in patients with resting LVOT gradient <50 mm Hg (40). The only limitation is that not all patients with HCM and controls are able to perform stress exercises. Patients with HCM usually present with dynamic LVOT gradient, arrhythmias, and mitral regurgitation. However, for asymptomatic carriers with pathogenic sarcomere mutations, the exercise stress test is potentially an advantageous method for unmasking subclinical diseases, such as impairment in diastolic function and dynamic outflow tract obstruction in the absence of a pathologically hypertrophied LV. The exercise stress test results presented here seem to have to accurately detect early changes in cardiac function, and the testing procedure was shown to be safe for enhancing and visualizing mid-range changes in cardiac function in variant carriers (41). Based on the results, asymptomatic *MYBPC3*^Δ25bp^ carriers do have sublevel phenotype during exercise, which is masked under baseline conditions. These data further indicate that exercise testing may assist in risk stratification for the presence of any cardiac events as previously described (41–43).

Hypertension, diabetes mellitus, coronary artery disease, and other comorbid conditions were known to confound the phenotypic effects of cardiomyopathy and cardiac dysfunction. Initial studies have shown that the *MYBPC3*^Δ25bp^ variant is associated with HCM (29), but then it was also determined to be associated with dilated and restrictive cardiomyopathies and HF (26). Later, it was determined that *MYBPC3*^Δ25bp^ is significantly associated with LV dysfunction secondary to coronary artery disease (39, 44). After myocardial infarction, results from these studies have suggested that patients with *MYBPC3*^Δ25bp^ show poor cardiac remodeling and recovery from the damage. This could be explained in several ways. First, cMyBP-C is sensitive to proteolysis during myocardial infarction and could be a potential earlier biomarker for heart attack (45–47). Thus, patients with *MYBPC3*^Δ25bp^ may not have enough wild-type cMyBP-C turnover, resulting in haploinsufficiency (48). The failure to compensate for the loss of wild-type during myocardial infarction finally results in poor cardiac remodeling. Second, when *MYBPC3*^Δ25bp^ carriers undergo myocardial infarction, it is possible that initial damage hinders quick recovery by the poison polypeptide effect (49). In the present study, we provided subjects with a questionnaire asking for information pertaining to their demographics, family history, medical history, and current and past medications. Using this as our guide, we then excluded any *MYBPC3*^Δ25bp^ carriers who were presented with such secondary risk factors, thus eliminating them from participation in the stress echo study. This means that the outcome of the stress echo was based solely on the presence of *MYBPC3*^Δ25bp^ and its effects on cardiac function. Collectively, results from the present study suggest LV hypercontractility under exercise stress conditions with evidence of diastolic stiffening and impairment among male asymptomatic *MYBPC3*^Δ25bp^ carriers. As such, these results also suggest the presence of some subclinical pathology in *MYBPC3*^Δ25bp^ carriers under exercise stress conditions. Based on the presence of HCM in transgenic mice expressing *MYBPC3*^Δ25bp^ mRNA (49) and subclinical cardiomyopathy phenotype in carriers (26), *MYBPC3*^Δ25bp^ can, therefore, be considered a potential risk allele for abnormal diastolic function and cardiomyopathy.

Complementary DNA sequencing of mRNA isolated from the biopsy of an *MYBPC3*^Δ25bp^-positive patient confirmed the presence of exon 33 skipping *in vivo* (26, 29). Exon 33 skipping results in the loss of 62 amino acids, resulting in a modified C10 domain of cMyBP-C (13). Exon 33 skipping also moves the stop codon to the 3′ untranslated region (UTR), adding a novel 55 amino acids in this newly modified C10 domain at the carboxyl terminus (cMyBP-C^ΔC10^) (49). However, if the C10 domain is modified, or truncated, then cMyBP-C will not properly localize in the sarcomere (50–52). The repercussions of this event can involve pathogenicity with the onset of HCM.

In fact, we have previously demonstrated that the presence of the *MYBPC3*^Δ25bp^ variant could lead to exon 33 skipping during transcription (26, 29). We next determined that the *MYBPC3*^Δ25bp^ variant in the presence of exon 33 skipping is pathogenic, both *in vitro* (46) and *in vivo* (49). Using a transgenic mouse model, we demonstrated that the expression of cMyBP-C^ΔC10^ is sufficient to cause HCM. Based on this observation, we propose that haploinsufficiency increases the disordered relaxed state (DRX) and decreases the super relaxed state (SRX) of myosin, leading to hypercontraction and HCM (53, 54). However, the molecular mechanism underlying these phenomena is yet to be validated in a humanized knock-in *MYBPC3*^Δ25bp^ mouse model that shows normal cardiac function under baseline conditions (55). Thus, future studies will involve defining the pathogenicity of *MYBPC3*^Δ25bp^ in a humanized knock-in mouse model in the presence of a comorbidity or acute insult.

### Study Limitation

This study is limited by its single-center nature, small cohort size, and inclusion of only men. Although HCM has equal distribution in both sexes (4, 56), women are more likely to remain undiagnosed (4); therefore, including female carriers and evaluating gender-specific manifestations under exercise stress conditions are strongly recommended. However, among those contacted for this study, male subjects were more willing to perform TTE and/or BSE, accounting for the disparity. We were also limited by the coronavirus disease-2019 (COVID-19), which resulted in a delay in recruitment and the need to follow health and safety protocols, such as wearing a mask during stress exercise. While the present study is valuable for its novel outcome, it is, by design, only a pilot study, so further, larger studies are needed.

## Author's Note

We presented the current data at the American Heart Association Scientific Sessions on November 12, 2020 (abstract # 13481) entitled “South Asian-specific *MYBPC3*^Δ25bp^ Intronic Deletion Carriers Demonstrate Hypercontractility and Impaired Diastolic Function Under Exercise Stress” (Circulation. 2020;142:A13481; https://doi.org/10.1161/circ.142.suppl_3.13481).

## Data Availability Statement

The original contributions presented in the study are included in the article/supplementary material, further inquiries can be directed to the corresponding author/s.

## Ethics Statement

The studies involving human participants were reviewed and approved by Prevalence study: Loyola University Chicago IRB# LU207815 and 207359, Chicago, Illinois and University of Cincinnati IRB# 2016-7580, Cincinnati, Ohio. Genotype-phenotype study: University of Cincinnati IRB# 2016-4948, Cincinnati, Ohio. The patients/participants provided their written informed consent to participate in this study.

## Author Contributions

SB, SV, EM, and SS: conceptualization. SB, RS, SG, MP, KS, RRS, MK, DH, JR, and EM: data curation. SB, RS, SG, KS, MK, RJ, DH, JR, and EM: formal analysis. RB and SS: funding acquisition. SB, RS, SG, MP, MK, DH, JR, EM, and SS: investigation. SB, SV, MP, RJ, DH, JR, EM, and SS: methodology. DH, EM, and SS: project administration. RS, KS, RRS, RJ, DH, JR, RB, EM, and SS: resources. DH, EM, and SS: supervision. RJ, JR, and RB: validation. SB, MP, and SS: writing the original draft. SB, SS, MP, RJ, DH, JR, RB, EM, and SS: writing, reviewing, and editing. All authors contributed to the article and approved the submitted version.

## Funding

This research was supported by Heart, Lung, and Vascular Institute startup funding at the College of Medicine, University of Cincinnati. The investigators were further supported by the National Institutes of Health grants R01 AR078001 (SS), R01 HL130356 (SS), R01 HL105826 (SS), R38 HL155775 (SS), R01 HL143490 (SS), U01 HL131914 (EM), and R01 HL128075 (EM), and the American Heart Association 2019 Institutional Undergraduate Student (19UFEL34380251, SS), Cardiovascular Genome-Phenome Study (15CVGPSD27020012, SS, MP, and EM), Catalyst (17CCRG33671128, SS, MP, and EM), Transformation (19TPA34830084, SS), Career Development (189CDA34110460, MP), and Postdoctoral Training Fellowship (17POST33630157, SV) awards, and the PLN Foundation (PLN crazy idea, SS). RRS was supported by an Amgen Postdoctoral Fellowship.

## Conflict of Interest

SS provides consulting and collaborative research studies to the Leducq Foundation (CURE-PLAN), Red Saree Inc., Greater Cincinnati Tamil Sangam, Pfizer, Novo Nordisk, AstraZeneca, MyoKardia, Merck, and Amgen. EM serves as a consultant to AstraZeneca, Amgen, Pfizer, Tenaya Therapeutics, and Invitae. RB serves on scientific advisory boards for Janssen and Basking Biosciences and DSMB Committees for Ionis Pharmaceuticals, Akcea Therapeutics, and Novartis. These activities are unrelated to the content of this work. RRS has been a postdoctoral fellow of Amgen, starting from June 2019, and performs research at the University of Cincinnati. The remaining authors declare that the research was conducted in the absence of any commercial or financial relationships that could be construed as a potential conflict of interest.

## Publisher's Note

All claims expressed in this article are solely those of the authors and do not necessarily represent those of their affiliated organizations, or those of the publisher, the editors and the reviewers. Any product that may be evaluated in this article, or claim that may be made by its manufacturer, is not guaranteed or endorsed by the publisher.

## References

[1] Maron BJ Gardin JM Flack JM Gidding SS Kurosaki TT Bild DE. Prevalence of hypertrophic cardiomyopathy in a general population of young adults. Echocardiographic analysis of 4111 subjects in the CARDIA Study Coronary Artery Risk Development in (Young) Adults. Circulation. (1995) 92:785–9. 10.1161/01.CIR.92.4.785 7641357

[2] Maron BJ Peterson EE Maron MS Peterson JE. Prevalence of hypertrophic cardiomyopathy in an outpatient population referred for echocardiographic study. Am J Cardiol. (1994) 73:577–80. 10.1016/0002-9149(94)90337-9 8147304

[3] Semsarian C Ingles J Maron MS Maron BJ. New perspectives on the prevalence of hypertrophic cardiomyopathy. J Am Coll Cardiol. (2015) 65:1249–54. 10.1016/j.jacc.2015.01.019 25814232

[4] Ommen SR Mital S Burke MA Day SM Deswal A Elliott P. 2020 AHA/ACC Guideline for the diagnosis and treatment of patients with hypertrophic cardiomyopathy: a report of the American College of Cardiology/American Heart Association Joint Committee on Clinical Practice Guidelines. Circulation. (2020) 142:e533–57. 10.1161/CIR.0000000000000938 33215938

[5] Watkins H MacRae C Thierfelder L Chou YH Frenneaux M McKenna W. A disease locus for familial hypertrophic cardiomyopathy maps to chromosome 1q3. Nat Genet. (1993) 3:333–7. 10.1038/ng0493-333 7981753

[6] Watkins H Conner D Thierfelder L Jarcho JA MacRae C McKenna WJ. Mutations in the cardiac myosin binding protein-C gene on chromosome 11 cause familial hypertrophic cardiomyopathy. Nat Genet. (1995) 11:434–7. 10.1038/ng1295-434 7493025

[7] Bonne G Carrier L Bercovici J Cruaud C Richard P Hainque B. Cardiac myosin binding protein-C gene splice acceptor site mutation is associated with familial hypertrophic cardiomyopathy. Nat Genet. (1995) 11:438–40. 10.1038/ng1295-438 7493026

[8] Carrier L Bonne G Schwartz K. Cardiac Myosin-binding protein C and hypertrophic cardiomyopathy. Trends Cardiovasc Med. (1998) 8:151–7. 10.1016/S1050-1738(97)00144-8 21235926

[9] Spirito P Seidman CE McKenna WJ Maron BJ. The management of hypertrophic cardiomyopathy. N Engl J Med. (1997) 336:775–85. 10.1056/NEJM199703133361107 9052657

[10] Harris SP Lyons RG Bezold KL. In the thick of it: HCM-causing mutations in myosin binding proteins of the thick filament. Circ Res. (2011) 108:751–64. 10.1161/CIRCRESAHA.110.231670 21415409 PMC3076008

[11] Gersh BJ Maron BJ Bonow RO Dearani JA Fifer MA Link MS. 2011 ACCF/AHA guideline for the diagnosis and treatment of hypertrophic cardiomyopathy: executive summary: a report of the American College of Cardiology Foundation/American Heart Association Task Force on Practice Guidelines. Circulation. (2011) 124:2761–96. 10.1161/CIR.0b013e318223e230 22068435

[12] Barefield D Kumar M de Tombe PP Sadayappan S. Contractile dysfunction in a mouse model expressing a heterozygous MYBPC3 mutation associated with hypertrophic cardiomyopathy. Am J Physiol Heart Circ Physiol. (2014) 306:H807–15. 10.1152/ajpheart.00913.2013 24464755 PMC3949045

[13] Kuster DW Sadayappan S. MYBPC3's alternate ending: consequences and therapeutic implications of a highly prevalent 25 bp deletion mutation. Pflugers Arch. (2014) 466:207–13. 10.1007/s00424-013-1417-7 24327208 PMC3946836

[14] Previs MJ Beck Previs S Gulick J Robbins J Warshaw DM. Molecular mechanics of cardiac myosin-binding protein C in native thick filaments. Science. (2012) 337:1215–8. 10.1126/science.1223602 22923435 PMC3561468

[15] Morita H Rehm HL Menesses A McDonough B Roberts AE Kucherlapati R. Shared genetic causes of cardiac hypertrophy in children and adults. N Engl J Med. (2008) 358:1899–908. 10.1056/NEJMoa075463 18403758 PMC2752150

[16] Niimura H Bachinski LL Sangwatanaroj S Watkins H Chudley AE McKenna W. Mutations in the gene for cardiac myosin-binding protein C and late-onset familial hypertrophic cardiomyopathy. N Engl J Med. (1998) 338:1248–57. 10.1056/NEJM199804303381802 9562578

[17] Moolman JA Reith S Uhl K Bailey S Gautel M Jeschke B. A newly created splice donor site in exon 25 of the MyBP-C gene is responsible for inherited hypertrophic cardiomyopathy with incomplete disease penetrance. Circulation. (2000) 101:1396–402. 10.1161/01.CIR.101.12.1396 10736283

[18] Adalsteinsdottir B Teekakirikul P Maron BJ Burke MA Gudbjartsson DF Holm H. Nationwide study on hypertrophic cardiomyopathy in Iceland: evidence of a MYBPC3 founder mutation. Circulation. (2014) 130:1158–67. 10.1161/CIRCULATIONAHA.114.011207 25078086

[19] Helms AS Davis FM Coleman D Bartolone SN Glazier AA Pagani F. Sarcomere mutation-specific expression patterns in human hypertrophic cardiomyopathy. Circ Cardiovasc Genet. (2014) 7:434–43. 10.1161/CIRCGENETICS.113.000448 25031304 PMC4254656

[20] Michels M Soliman OI Phefferkorn J Hoedemaekers YM Kofflard MJ Dooijes D. Disease penetrance and risk stratification for sudden cardiac death in asymptomatic hypertrophic cardiomyopathy mutation carriers. Eur Heart J. (2009) 30:2593–8. 10.1093/eurheartj/ehp306 19666645

[21] Page SP Kounas S Syrris P Christiansen M Frank-Hansen R Andersen PS. Cardiac myosin binding protein-C mutations in families with hypertrophic cardiomyopathy: disease expression in relation to age, gender, and long term outcome. Circ Cardiovasc Genet. (2012) 5:156–66. 10.1161/CIRCGENETICS.111.960831 22267749

[22] Niimura H Patton KK McKenna WJ Soults J Maron BJ Seidman JG. Sarcomere protein gene mutations in hypertrophic cardiomyopathy of the elderly. Circulation. (2002) 105:446–51. 10.1161/hc0402.102990 11815426

[23] Maron BJ Shirani J Poliac LC Mathenge R Roberts WC Mueller FO. Sudden death in young competitive athletes. Clinical, demographic, and pathological profiles. JAMA. (1996) 276:199–204. 10.1001/jama.1996.03540030033028 8667563

[24] Klues HG Schiffers A Maron BJ. Phenotypic spectrum and patterns of left ventricular hypertrophy in hypertrophic cardiomyopathy: morphologic observations and significance as assessed by two-dimensional echocardiography in 600 patients. J Am Coll Cardiol. (1995) 26:1699–708. 10.1016/0735-1097(95)00390-8 7594106

[25] Maron BJ. Hypertrophic cardiomyopathy: an important global disease. Am J Med. (2004) 116:63–5. 10.1016/j.amjmed.2003.10.012 14706671

[26] Dhandapany PS Sadayappan S Xue Y Powell GT Rani DS Nallari P. A common MYBPC3 (cardiac myosin binding protein C) variant associated with cardiomyopathies in South Asia. Nat Genet. (2009) 41:187–91. 10.1038/ng.309 19151713 PMC2697598

[27] Viswanathan SK Puckelwartz MJ Mehta A Ramachandra CJA Jagadeesan A Fritsche-Danielson R. Association of cardiomyopathy with MYBPC3 D389V and MYBPC3Delta25bpIntronic deletion in South Asian Descendants. JAMA Cardiol. (2018) 3:481–8. 10.1001/jamacardio.2018.0618 29641836 PMC6054452

[28] Arif M Nabavizadeh P Song T Desai D Singh R Bazrafshan S. Genetic, clinical, molecular, and pathogenic aspects of the South Asian-specific polymorphic MYBPC3(Delta25bp) variant. Biophys Rev. (2020) 12:1065–84. 10.1007/s12551-020-00725-1 32656747 PMC7429610

[29] Waldmuller S Sakthivel S Saadi AV Selignow C Rakesh PG Golubenko M. Novel deletions in MYH7 and MYBPC3 identified in Indian families with familial hypertrophic cardiomyopathy. J Mol Cell Cardiol. (2003) 35:623–36. 10.1016/S0022-2828(03)00050-6 12788380

[30] Sadayappan S Puckelwartz MJ McNally EM. South Asian-specific MYBPC3(Delta25bp) intronic deletion and its role in cardiomyopathies and heart failure. Circ Genom Precis Med. (2020) 13:e002986. 10.1161/CIRCGEN.120.002986 32543992 PMC7301913

[31] Harper AR Bowman M Hayesmoore JBG Sage H Salatino S Blair E. Reevaluation of the South Asian MYBPC3(Delta25bp) intronic deletion in hypertrophic cardiomyopathy. Circ Genom Precis Med. (2020) 13:e002783. 10.1161/CIRCGEN.119.002783 32163302 PMC7299222

[32] Puckelwartz MJ Pesce LL Nelakuditi V Dellefave-Castillo L Golbus JR Day SM. Supercomputing for the parallelization of whole genome analysis. Bioinformatics. (2014) 30:1508–13. 10.1093/bioinformatics/btu071 24526712 PMC4029034

[33] Richards S Aziz N Bale S Bick D Das S Gastier-Foster J. ACMG laboratory quality assurance committee. standards and guidelines for the interpretation of sequence variants: a joint consensus recommendation of the American College of Medical Genetics and Genomics and the Association for Molecular Pathology. Genet Med. (2015) 5:405–24. 10.1038/gim.2015.30 25741868 PMC4544753

[34] Ho CY Carlsen C Thune JJ Havndrup O Bundgaard H Farrohi F. Echocardiographic strain imaging to assess early and late consequences of sarcomere mutations in hypertrophic cardiomyopathy. Circ Cardiovasc Genet. (2009) 2:314–21. 10.1161/CIRCGENETICS.109.862128 20031602 PMC2773504

[35] Michels M Soliman OI Kofflard MJ Hoedemaekers YM Dooijes D Majoor-Krakauer D. Diastolic abnormalities as the first feature of hypertrophic cardiomyopathy in Dutch myosin-binding protein C founder mutations. JACC Cardiovasc Imaging. (2009) 2:58–64. 10.1016/j.jcmg.2008.08.003 19356534

[36] Olivotto I Cecchi F Poggesi C Yacoub MH. Patterns of disease progression in hypertrophic cardiomyopathy: an individualized approach to clinical staging. Circ Heart Fail. (2012) 5:535–46. 10.1161/CIRCHEARTFAILURE.112.967026 22811549

[37] Bashyam MD Purushotham G Chaudhary AK Rao KM Acharya V Mohammad TA. A low prevalence of MYH7/MYBPC3 mutations among familial hypertrophic cardiomyopathy patients in India. Mol Cell Biochem. (2012) 360:373–82. 10.1007/s11010-011-1077-x 21959974

[38] Simonson TS Zhang Y Huff CD Xing J Watkins WS Witherspoon DJ. Limited distribution of a cardiomyopathy-associated variant in India. Ann Hum Genet. (2010) 74:184–8. 10.1111/j.1469-1809.2010.00561.x 20201939 PMC2901538

[39] Srivastava A Garg N Mittal T Khanna R Gupta S Seth PK. Association of 25 bp deletion in MYBPC3 gene with left ventricle dysfunction in coronary artery disease patients. PLoS ONE. (2011) 6:e24123. 10.1371/journal.pone.0024123 21915287 PMC3168477

[40] Sorensen LL Liang HY Pinheiro A Hilser A Dimaano V Olsen NT. Safety profile and utility of treadmill exercise in patients with high-gradient hypertrophic cardiomyopathy. Am Heart J. (2017) 184:47–54. 10.1016/j.ahj.2016.10.010 27892886 PMC5322224

[41] Desai MY Bhonsale A Patel P Naji P Smedira NG Thamilarasan M. Exercise echocardiography in asymptomatic HCM: exercise capacity, and not LV outflow tract gradient predicts long-term outcomes. JACC Cardiovasc Imaging. (2014) 7:26–36. 10.1016/j.jcmg.2013.08.010 24290569

[42] Mora S Redberg RF Cui Y Whiteman MK Flaws JA Sharrett AR. Ability of exercise testing to predict cardiovascular and all-cause death in asymptomatic women: a 20-year follow-up of the lipid research clinics prevalence study. JAMA. (2003) 290:1600–7. 10.1001/jama.290.12.1600 14506119

[43] Myers J Prakash M Froelicher V Do D Partington S Atwood JE. Exercise capacity and mortality among men referred for exercise testing. N Engl J Med. (2002) 346:793–801. 10.1056/NEJMoa011858 11893790

[44] Kumar S Mishra A Srivastava A Bhatt M Garg N Agarwal SK. Role of common sarcomeric gene polymorphisms in genetic susceptibility to left ventricular dysfunction. J Genet. (2016) 95:263–72. 10.1007/s12041-016-0623-4 27350668

[45] Yogeswaran A Troidl C McNamara JW Wilhelm J Truschel T Widmann L. The C0-C1f region of cardiac myosin binding protein-C induces pro-inflammatory responses in fibroblasts via TLR4 signaling. Cells. (2021) 10:1326. 10.3390/cells10061326 34073556 PMC8230336

[46] Kuster DW Govindan S Springer TI Martin JL Finley NL Sadayappan S. A hypertrophic cardiomyopathy-associated MYBPC3 mutation common in populations of South Asian descent causes contractile dysfunction. J Biol Chem. (2015) 290:5855–67. 10.1074/jbc.M114.607911 25583989 PMC4342493

[47] Chen XJ Zhang W Bian ZP Wang ZM Zhang J Wu HF. Cardiac myosin-binding protein c release profile after cardiac surgery in intensive care unit. Ann Thorac Surg. (2019) 108:1195–201. 10.1016/j.athoracsur.2019.03.072 31034826

[48] Glazier AA Thompson A Day SM. Allelic imbalance and haploinsufficiency in MYBPC3-linked hypertrophic cardiomyopathy. Pflugers Arch. (2019) 471:781–93. 10.1007/s00424-018-2226-9 30456444 PMC6476680

[49] Kuster DWD Lynch TL Barefield DY Sivaguru M Kuffel G Zilliox MJ. Altered C10 domain in cardiac myosin binding protein-C results in hypertrophic cardiomyopathy. Cardiovasc Res. (2019) 115:1986–97. 10.1093/cvr/cvz111 31050699 PMC6872972

[50] Moolman-Smook J Flashman E de Lange W Li Z Corfield V Redwood C. Identification of novel interactions between domains of Myosin binding protein-C that are modulated by hypertrophic cardiomyopathy missense mutations. Circ Res. (2002) 91:704–11. 10.1161/01.RES.0000036750.81083.83 12386147

[51] McConnell BK Jones KA Fatkin D Arroyo LH Lee RT Aristizabal O. Dilated cardiomyopathy in homozygous myosin-binding protein-C mutant mice. J Clin Invest. (1999) 104:1235–44. 10.1172/JCI7377 10545522 PMC409819

[52] Hossain MB Elbeck Z Siga H Knoll R. Myosin binding protein-C and hypertrophic cardiomyopathy: role of altered C10 domain. Cardiovasc Res. (2019) 115:1943–5. 10.1093/cvr/cvz167 31263890

[53] McNamara JW Li A Lal S Bos JM Harris SP van der Velden J. MYBPC3 mutations are associated with a reduced super-relaxed state in patients with hypertrophic cardiomyopathy. PLoS ONE. (2017) 12:e0180064. 10.1371/journal.pone.0180064 28658286 PMC5489194

[54] Toepfer CN Wakimoto H Garfinkel AC McDonough B Liao D Jiang J. (2019). Hypertrophic cardiomyopathy mutations in MYBPC3 dysregulate myosin. Sci. Transl. Med. 11:eaat1199. 10.1126/scitranslmed.aat1199 30674652 PMC7184965

[55] McNamara JW Schwanekamp JA Patel PN Viswanathan SK Bohlooly M Madeyski-Bengtson K. The highly prevalent 25bp intronic deletion of MYBPC3 is benign under baseline conditions. Circ Res. (2019) 125:772. 10.1161/res.125.suppl_1.77226078378

[56] Olivotto I Maron MS Adabag AS Casey SA Vargiu D Link MS. Gender-related differences in the clinical presentation and outcome of hypertrophic cardiomyopathy. J Am Coll Cardiol. (2005) 46:480–7. 10.1016/j.jacc.2005.04.043 16053962

